# Intussusception caused by an inverted colonic diverticulum: a case report

**DOI:** 10.1186/s13256-018-1652-9

**Published:** 2018-04-27

**Authors:** Bei Zhang, Jiping Wang, Xiaoguang Li, Zhuo Wang, Yangjiao Zhang, Hao Yang

**Affiliations:** 1 Department of Radiology, First Hospital of Jilin University, Changchun, China; 2 Department of Gastrointestinal Surgery, First Hospital of Jilin University, No.71, Xinmin Street, Changchun, 130021 China

**Keywords:** Emergency operation, Intussusception, Inverted colonic diverticulum

## Abstract

**Background:**

Intussusception is an unusual disorder among the complications of diverticula in adults. This study aimed to report intussusception due to an inverted colonic diverticulum. Such a large inverted colonic diverticulum has rarely been reported.

**Case presentation:**

A 62-year-old Chinese woman presented to the First Hospital of Jilin University with abdominal pain, vomiting, and bloody stool. Preoperative computed tomography imaging indicated intussusception. The intraoperative diagnosis was consistent with postoperative pathology. Our patient was diagnosed as having an inverted colonic diverticulum near the ileocecal valve with intussusception and intestinal necrosis.

**Conclusion:**

Although inverted colonic diverticulum is extremely rare, it should also be considered among the causative factors of intussusception.

## Background

Colonic diverticulum seldom has clinical symptoms [1]. When colonic diverticulum is associated with complications, surgical treatment may be considered [2]. Intussusception is one of the emergency situations. Among the most reported complications, intussusception was described occasionally, but an inverted colonic diverticulum (ICD) as large as the one reported in the present study has rarely been described. The correct preoperative diagnosis of intussusception due to ICD has rarely been made [3]. This study aimed to present the case of an ICD complicated by ileocolonic intussusception in a Chinese elderly woman successfully treated surgically.

## Case presentation

**Fig. 1:**
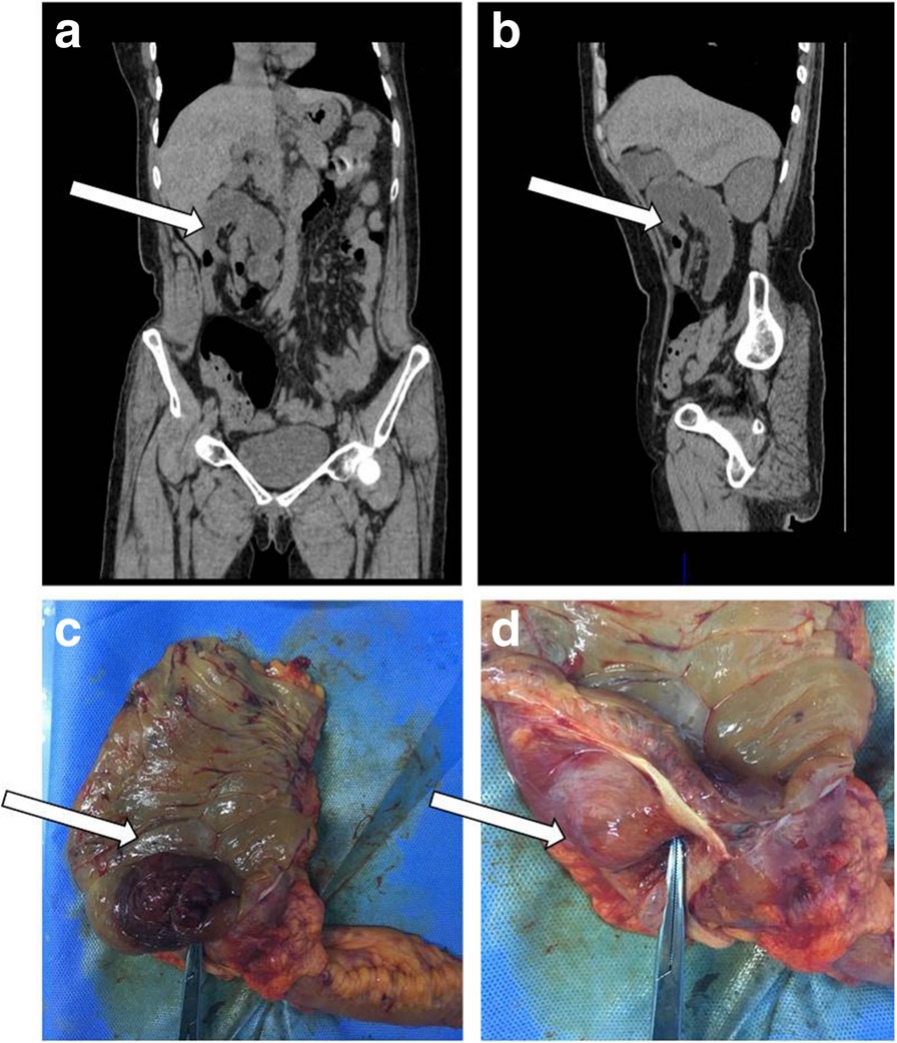
Computed tomography imaging and gross sample. **a** and **b** A computed tomography scan showed the target sign (*white arrow*) in the right abdomen. Colonic diverticulum entered the right colon. **c** and **d** Gross specimen showed the entry of colonic diverticulum (*white arrow*) into the ascending colon

A 62-year-old Chinese woman presented to the First Hospital of Jilin University with complaints of abdominal pain, vomiting, and bloody stool for 24 hours. On admission, her general condition was good. Her temperature was 37.1 °C, respiratory rate was 18 times per minute, and heart rate was 80 beats per minute. She did not have cutaneous or scleral icterus. No superficial tumescent lymph nodes were observed. A physical examination showed distended abdomen with no stomach outline, peristalsis, and varicose veins. Abdominal breathing was slightly limited. Abdominal tenderness could be detected in her right abdomen accompanied by rebound tenderness and muscle tension. A palpable mass could be detected in her right lower abdomen. Liver, spleen, and gallbladder could not be touched below the costal arch. Murphy’s sign and shifting dullness were negative. Bowel sound was found to be approximately 7 beats per minute with gurgling. A neurological physical examination had no significant findings. A physical examination of her heart and chest was normal. She was a housewife in a small city with no special family history. She had a history of hypertension for 10 years, and the use of captopril helped maintain a satisfactory level of blood pressure. She denied a history of hepatitis, tuberculosis, and diabetes. Also, no history of drug allergy, surgeries, tobacco smoking, and long-term alcohol consumption was reported. The results of complete blood count, liver function, and blood biochemical indexes are shown in Tables 1 and 2. The results of coagulation markers of hepatitis B virus, hepatitis C virus, syphilis, and human immunodeficiency virus were found to be negative. Abdominal computed tomography (CT) was performed, which showed some intestinal canals and mesentery entering into the right colon. The CT scan showed a target sign suggesting intussusception in her right abdomen (Fig. 1a, b).

**Table 1:** Complete blood count before and after surgery

Items	Unit	Before surgery	First day after surgery	Second day after surgery
Red blood cell (RBC)	× 10^12^/L	4.65	3.96	3.80
Hemoglobin (HGB)	g/L	140	118	117
Hematocrit (HCT)	L/L	0.411	0.353	0.352
Mean corpuscular volume (MCV)	fL	88.4	89.1	92.6
Mean corpuscular hemoglobin (MCH)	pg	30.1	29.8	30.8
Mean corpuscular hemoglobin concentration (MCHC)	g/L	341	334	332
Platelet (PLT)	× 10^9^/L	216	196	167
White blood cell (WBC)	× 10^9^/L	9.40	17.21	10.62
Neutrophil count (NE)	× 10^9^/L	8.12	14.45	8.98
Lymphocyte count (LY)	× 10^9^/L	0.95	1.57	0.79
Monocyte count (MO)	× 10^9^/L	0.32	1.15	0.82
Eosinophil count (EO)	× 10^9^/L	0.01	0.04	0.01
Neutrophil percentage (NE%)		0.86	0.84	0.85
Lymphocyte percentage (LY%)		0.10	0.09	0.07
Monocyte percentage (MO%)		0.03	0.07	0.08
Eosinophil percentage (EO%)		0.00	0.00	0.00

**Table 2:** Blood biochemical examination

Items	Unit	Before surgery	First day after surgery
Alanine aminotransferase (ALT)	U/L	17.1	
Alkaline phosphatase (ALP)	U/L	51.7	
Total protein (TP)	g/L	67.7	
Albumin (ALB)	g/L	41.9	
Globulin (GLO)	g/L	25.8	
Albumin/Globulin (A/G)		1.62	
Total bilirubin in serum (TBIL)	μmol/L	12.4	
Direct bilirubin in serum (DBIL)	μmol/L	2.1	
Indirect bilirubin in serum (IBIL)	μmol/L	10.3	
Blood urea nitrogen (BUN)	mmol/L		
Serum creatinine (Cr)	μmol/L	47.7	52.0
Urea	mmol/L	4.90	5.21
K^+^	mmol/L	3.97	3.64
Na+	mmol/L	134.9	131.6
Cl^−^	mmol/L	100.1	99.5
Ca^2+^	mmol/L	2.16	1.93

**Fig. 2:**
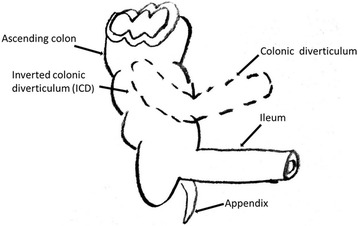
Schematic image of the intussusception caused by an inverted colonic diverticulum. *ICD* inverted colonic diverticulum

Surgical exploration revealed that the ICD entered her ascending colon with edema (Figs. 1c, d and 2). The diverticulum was suspected of blood supply deficiency. No obvious abnormalities were found in the remaining colon and rectum. Intussusception was diagnosed during the operation. Right hemicolectomy was performed under laparoscopy.

**Fig. 3:**
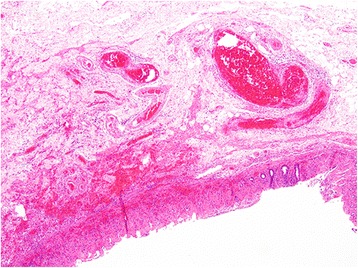
Histology of inverted colonic diverticulum. The true diverticulum and the full thickness of the bowel wall from the mucosa to the serosa in a low-power field (hematoxylin and eosin, × 4)

A histopathological examination showed that the ICD was on the side of ascending colon, causing ileocolonic intussusception with intestinal necrosis (Fig. 3). The diameter was approximately 3.8 cm.

Cefmenoxime hydrochloride injection (2 g) was used before and after the surgery to prevent infection. Our patient quickly recovered and was discharged 7 days after the surgery without any complications. She had a telephonic follow-up 10 months after discharge from our hospital. She had no apparent symptoms after the surgery. Serological and imaging examinations were performed in a local hospital, and the results were negative.

## Discussion

Colonic diverticular disease is a common condition. However, it has become increasingly difficult to ignore a singular relatively large diverticulum. A previous study reported giant ascending colonic diverticulum with intussusception [3]. The diameter of the diverticulum described in that report was larger than 4 cm, but the diverticulum in the present study was not as large as that giant colonic diverticulum. In the present study, intussusception was caused by an ICD with a diameter of 3.8 cm.

Intussusception refers to the insertion of an intestinal tube into the contiguous lumen, usually leading to intestinal obstruction and ischemia [4]. Intussusception in adults accounts for 1–3% of intestinal obstruction in adults [5]. The etiology of more than 90% of adult intussusceptions has been reported [6]. However, the preoperative diagnosis of adult intussusception still remains difficult. Abdominal CT is usually used in the preoperative evaluation. Surgery is the recommended treatment.

ICD is rare. The incidence rate of ICD is approximately 0.7% [7]. The smaller ICD tends to be indistinguishable from colonic polyps. Endoscopic biopsy or polypectomy can be dangerous in such a situation [7]. The water jet deformation sign is thought to be a safer and more reliable method compared with biopsy forceps and air insufflation [8]. The differential diagnosis includes cecal volvulus, a duplication cyst or giant Meckel diverticulum, and pneumatosis cystoides intestinalis.

The lesion in the patient in this study was unusual. It occurred in the colon close to the ileocecal valve. The diameter was 3.8 cm, not larger than 4.0 cm, and the lesion was not located in the sigmoid colon. It could not be defined as a giant colonic diverticulum because the diameter of a giant colonic diverticulum needs to be larger than 4 cm, more often occurring in the sigmoid colon [9]. The mechanism of the formation of large diverticula is not fully known. One possibility is the ball-valve mechanism. A narrow passage between the normal bowel and the diverticulum makes the diverticulum larger [10]. Although the size of the lesion reported in this study did not reach 4.0 cm, the evolutionary process of a giant colonic diverticulum could not be excluded. Any intestinal condition that changes the normal pattern of peristalsis increases the risk of intussusception. Colonic diverticulum as a rare cause of adult intussusception was shown in this study. A previous study retrospectively reviewed 16 years of data from patients with intussusception and found that 77.3% of cases were related to a tumor, 73.5% of which were malignant. Among these, 11.3% occurred in a postoperative setting and 11.3% were idiopathic [11]. The intussusception due to ICD in the present case is a rare event in adult patients. Although the exact mechanism of invagination is unknown in this case, it is speculated that the existence of colonic diverticulum caused abnormal peristalsis of the intestines. Furthermore, the diameter of the diverticulum was relatively large in size.

A previous study reported intussusception caused by a giant colonic diverticulum. A laparoscopic right hemicolectomy was performed for one-stage treatment [3]. Roch *et al.* reported that laparoscopic atypical colon wedge resection was safely performed, and it might be considered an alternative for extended resections of giant diverticula [12].

## Conclusion

Although such a large ICD is extremely rare, it should also be considered among the causative factors of intussusception.
